# A multimedia campaign to improve back beliefs in patients with non-specific low back pain: a process evaluation

**DOI:** 10.1186/s12891-017-1551-z

**Published:** 2017-05-18

**Authors:** Arnela Suman, Frederieke G. Schaafsma, Jiman Bamarni, Maurits W. van Tulder, Johannes R. Anema

**Affiliations:** 1 Amsterdam Public Health research institute, Department of Public and Occupational Health, VU University Medical Centre, PO Box 7057, 1007 MB Amsterdam, The Netherlands; 2 Department of Public and Occupational Health, Research Centre for Insurance Medicine, Collaboration between AMC-UMCG-UWV-VUmc, VU University medical centre, PO Box 7067, 1007 MB Amsterdam, The Netherlands; 3 Faculty of Earth & Life Sciences, Department of Health Sciences, Student Health Sciences at the VU University Amsterdam, De Boelelaan 1085, 1081 HV Amsterdam, The Netherlands; 4 Amsterdam Public Health research institute, Faculty of Earth & Life Sciences, Department of Health Sciences, VU University Amsterdam, De Boelelaan 1085, 1081 HV Amsterdam, The Netherlands

**Keywords:** Implementation, Guidelines as topic, Process Assessment (Health Care), Multimedia, eHealth, Low back pain, Attitude to Health, Patient Satisfaction

## Abstract

**Background:**

Low back pain (LBP) is one of the most prevalent and costly disorders worldwide. To reduce its burden in the Netherlands, implementation of a multidisciplinary guideline for LBP was supported by a multifaceted eHealth campaign for patients with LBP. The current study aims 1) to evaluate whether the implementation strategy was performed as planned; 2) to assess the feasibility, barriers and facilitators of the patient based eHealth campaign; 3) to gain insight into the satisfaction and experiences of patients with various ethnic backgrounds with the implementation strategy and to make a comparison between them; and 4) to explore the association between exposure to and satisfaction with the implementation strategy.

**Methods:**

This process evaluation was performed using the Linnan and Steckler framework, and used a mixed methods approach for data collection and analysis. The relationship between satisfaction of patients and exposure to the strategy was statistically examined. Semi-structured interviews were analysed using qualitative data analysis methods.

**Results:**

Two hundred and fourteen patients participated in the quantitative, and 44 in the qualitative analysis. Most were female and had a high level of education. Many patients did not use the campaign at all or only once, and those that did rated it as reasonable. Patient satisfaction with the campaign increased significantly with an increase in its use. Qualitative analysis showed that four main themes played a role in campaign rating and use: satisfaction with intervention components, perceived benefits of the intervention, usage of the intervention, and satisfaction with the medium used.

**Conclusion:**

This process evaluation showed that the eHealth campaign was used only by a small proportion of patients with non-specific LBP. It seemed that the campaign was offered to the patients too late, that the lay-out of the campaign did not meet patient needs, and that healthcare providers rarely discussed the campaign with their patients, while involvement of those providers seemed to improve trustworthiness of the campaign and increase its usage. It is important to invest effort into healthcare providers to motivate patients to use eHealth intervention and to tailor strategies better to the needs of users.

**Trial registration:**

Netherlands Trial Register (NTR): NTR4329. Registered December 20th, 2013.

## Background

Low back pain (LBP) is the leading cause of disability worldwide [1], and one of the most common conditions for which people in industrialised countries seek medical care [2]. While LBP represents an important economic burden on societies [3], in 85 to 95% of patients with LBP the pain cannot be attributed to a specific cause and is thus referred to as non-specific [4, 5]. Psychosocial risk factors, including stress, anxiety, depression, and pain coping strategies have been shown to play a substantial role in the aetiology and prognosis of non-specific LBP [4, 5]. A biopsychosocial approach for the treatment of LBP has been increasingly adopted [6]. This approach often exists of multidisciplinary programmes that encompass a combination of physical, psychological, educational, and work-related components for treatment [6]. A recent Cochrane review has shown that this approach to LBP rehabilitation is more effective than usual care and physical treatment in decreasing pain and disability, and improving work outcomes [6]. However, despite the widespread efforts to reduce the burden of LBP, it remains a highly prevalent and costly disorder.

In an attempt to reduce the burden of LBP in the Netherlands, in 2010 the ‘Multidisciplinary guideline for nonspecific low back pain’ was developed [7]. This guideline was implemented using a patient and professional multifaceted implementation strategy that is evaluated in a randomised controlled trial (RCT). Implementing guidelines into clinical practice can be challenging, with many factors influencing the uptake of these guidelines by health care professionals (HCPs) [8]. In order to better understand why implementation efforts do or do not result in sustainable changes, it is important that trials implementing guidelines also evaluate the implementation process [9]. Process evaluations can explain differences between observed and expected results, and can shed a light on the underlying mechanisms responsible for the observed effects [10]. These evaluations can thus be useful in improving existing strategies for the implementation of interventions, and they can be useful for the development of future implementation strategies [10, 11]. The current paper describes the process evaluation of the implementation strategy targeted at patients. A process evaluation of the HCP based strategy is reported elsewhere [12].

The goals of the current study were: 1) to evaluate whether the implementation strategy was performed as planned; 2) to assess the feasibility, barriers and facilitators of the patient based eHealth campaign; 3) to gain insight into the satisfaction and experiences of patients with various ethnic backgrounds with the implementation strategy and to make a comparison between them; and 4) to explore the association between exposure to and satisfaction with the strategy.

## Methods

This process evaluation was performed alongside a stepped-wedge RCT to test the cost-effectiveness of a multifaceted implementation strategy for the Dutch multidisciplinary guideline for nonspecific LBP. Details of the procedures and methods of the RCT, as well as details on the medical ethical review for this study have been reported elsewhere [13]. In this trial, multidisciplinary collaboration between HCPs was promoted by means of continuing medical education (CME) training sessions for health care professionals combined with a multimedia strategy for these HCPs. For patients with LBP, an interactive multifaceted eHealth strategy was developed.

### Study population

The study population for this process evaluation consisted primarily of LBP patients that participated in the trial. These patients were recruited through their participating HCPs. Patients in the trial allocated to the intervention group were invited to participate in this process evaluation by means of a quantitative evaluation questionnaire. Patients that completed the questionnaire were asked if they were also willing to participate in a qualitative evaluation of the intervention, and if so, these patients were personally contacted by telephone and e-mail. Furthermore, patients from the intervention group were invited to participate in the qualitative analysis by means of a call in monthly newsletters, and through calls via social media. The majority of the trial patients were native Dutch or western immigrants. Because 35% of Amsterdam citizens consists of non-western ethnic minorities of which the majority is of Turkish, Moroccan or Surinamese origin [14], extra effort was put into recruiting Turkish, Moroccan, and Surinamese LBP patients for this process evaluation to gain insight into their experiences and satisfactions as well. These patients did not participate in the trial but received access to parts of the intervention for the sole purpose of evaluating these parts of the process. These patients were recruited in several ways, including a personal invitation to evaluate the intervention by their HCP; via posters and take-away brochures in HCP practices and university buildings, and via posters in various meeting places in the city.

### Multifaceted implementation strategy

The multifaceted implementation strategy for patients consisted of a multimedia eHealth campaign that comprised of several components, described in more detail below.

#### Interactive website

An interactive website that included extensive written information about acute and chronic LBP was developed. The website provided information about the aetiology of LBP, the expected prognosis, and tips and tricks on how to self-manage LBP. Twelve short video-messages in which HCPs such as surgeons, doctors and therapists, and patients with LBP share their insights and advice on LBP and staying active, continuing or returning to work, and coping with LBP were also developed for this campaign. The video-messages were uploaded one by one on the website every 1 to 2 months. Furthermore, the website contained several physical therapy exercises in video and diagram format, and other downloadable documents such as information leaflets and self-help toolkits. Links to other informative websites and applications, video material, and reading material were also provided on the website. In order to prevent contamination by exposing patients from the control group to the intervention strategy, the website was protected by login. Eligible patients received login credentials after they had completed a baseline questionnaire at their inclusion in the study. The website was modified in such a way that only administrators could generate these login credentials. This was an extra effort to prevent undesirable exposure of the website to the control group.

#### Social media

The website contained a forum on which patients could chat with each other or with members of the research team, where they could ask questions, or share information. Also, a Facebook page and a Twitter account were opened to regularly post updates or interesting information about the study or about LBP in general. Except for the forum, which was part of the protected website, the social media options were open to anyone who had an account and wished to follow the research pages.

#### Monthly newsletters

Monthly newsletters were sent to the patients. The newsletters included updates on the study, news, reminders about new content on the website (e.g. new video-messages), tips and tricks for self-management of LBP, frequently asked questions, anecdotal items, and reminders to fill in follow-up questionnaires.

####  Translations

In order to involve the two largest local ethnic minority groups (e.g. Turkish and Moroccan immigrants) in the study, several items of the strategy were translated into Turkish and Arab languages. These included instructions and explanations of the follow-up questionnaires, instructions and an explanation of the main page of the website, and re-voiced copies of the video-messages.

### Data collection and analysis

This process evaluation was based on components developed by the Linnan and Steckler framework [11]. Using a mixed methods approach, quantitative and qualitative methods were applied to collect data on the components: recruitment, reach, dose delivered, dose received, satisfaction and barriers and facilitators (Table 1).

**Table 1:** Process evaluation components

Component	Definition	Data collection method
1. Recruitment	Procedures used to recruit patients	Description and minutes of recruitment procedure
2. Reach	Number of patients participating in the study as proportion of patients invited; and number of patients participating in the process evaluation as proportion of patients participating in the study	Minutes of research organisation
3. Dose delivered	Extent to which the protocol for implementation strategy was delivered by the intervention providers as planned	Minutes of research organisation
4. Dose received	Extent to which intervention was used by patients	Evaluation questionnaire Login registration data website Followers data social media
5. Satisfaction	Experiences of patients with intervention Patients’ overall satisfaction with intervention	Evaluation questionnaire Qualitative interviews
6. Barriers and facilitators	Barriers and facilitators for intervention use by patients	Qualitative interviews

#### Quantitative data collection

All patients that participated in the trial were sent an electronic evaluation questionnaire in the months January and February 2016. The questionnaire was designed to measure their usage of and satisfaction with the intervention. Table 2 shows the evaluation questionnaire items. Using regression modelling in IBM SPSS Statistics 22.0, the association between satisfaction with the intervention (outcome variable) and exposure to the intervention (i.e. usage of the website) was explored. To measure intervention use objectively, login data of the website and amount of followers on social media were registered.

**Table 2:** Items of evaluation questionnaire

Items	
1. How often did you visit the website? ^a^	11. Did you find the links useful?^c^
2. Did your HCP recommend this website to you?^b^	12. How often did you use the exercises provided?^f^
3. Did your HCP discuss this website with you?^b^	13. Did you find the exercises useful?^c^
4. Did you experience any added value from the website in addition to the treatment you received from your HCP?^c^	14. Were the advices applicable to you?^c^
5. Was the information on the website clear to you?^c^	15. Did you find the monthly newsletter useful?^c^
6. Did you find the website useful?^c^	16. Did you use social media for this study, if yes, which one(s)?^g^
7. How many video-messages have you watched?^d^	17. Did the website contribute to your recovery?^b^
8. Did you find the video-messages useful?^c^	18. If the website contributed to your recovery, which component(s) was/were the most helpful to you?^h^
9. On a scale from 0 (lowest) to 10 (highest), how would you rate your appreciation of the video-messages?^e^	19. Would you recommend this website to others with back pain?^b^
10. How often did you use the links provided?^f^	20. On a scale from 0 (lowest) to 10 (highest), how would you rate your appreciation of the website?^e^

^a^Never/Once/At least once per month/At least once, not every month

^b^Yes/No

^c^ No/A little/Yes

^d^None/One/Some (2–12)/All (12)

^e^0–10

^f^Never/Once/More than once

^g^Forum/Facebook/Twitter

^h^Information/Videos/Links/Exercises/Social media

#### Dose delivered

The dose delivered refers to the extent to which the strategy was delivered by the intervention providers according to protocol for the various implementation strategy components. Points were assigned to strategy components if they were delivered as planned, and the sum of component scores was used to calculate the overall dose delivered. Only the provision of video-messages and newsletters was protocolled, whereas the social media component was set up to provide ad hoc messages.

##### Video-messages

During 12 months, one video-message per one to two months was to be uploaded on the website. For each video-message that was timely delivered 1 point was scored, thus amounting to a maximum total of 12 points that could be scored for this component.

##### Monthly Newsletters

During 24 months, one newsletter was to be sent out to participating patients. For each newsletter that was timely delivered 1 point was scored, thus amounting to a maximum total of 24 points that could be scored for this component.

#### Qualitative data analysis

To gain more in-depth knowledge about the satisfaction and experiences of patients with the intervention, and to get insight into barriers and facilitators for the use of this intervention by patients, semi-structured, qualitative interviews were conducted among a subset of participating patients. Sampling of patients was guided by the willingness of the patients to participate in an interview, and interviews were conducted until redundancy was reached [15].

The topic lists for the interviews addressed the same items as the evaluation questionnaire, and was designed to elicit opinions about the various intervention components and the manner in which they were offered. Interviews were recorded and transcribed verbatim immediately after they had taken place. All interviews were analysed using a constant comparison approach in three subsequent steps. The first step was the fragmentation of transcripts into short, descriptive summaries (open coding). Subsequently, the fragments that were closely related to each other were grouped to create provisional themes (axial coding). In the third and last step, connection between the provisional themes was made to structure the data into meaningful entities [16]. To enhance the quality of analyses, one researcher coded all interviews, and two other researchers coded a random sample of interviews. Any disagreements in coding were resolved by consensus. The patients participating in this study were divided into four ethnic groups, and the satisfaction and experiences with the implementation strategy were compared between those groups.

## Results

Three hundred and 31 patients with LBP received the multifaceted intervention of this implementation study. A total of 214 (64.7%) patients completed the quantitative evaluation questionnaire for the current process evaluation, and 44 patients participated in the qualitative evaluation of the intervention.

### Quantitative results

#### Recruitment and reach

A total of 5203 patients with LBP were invited by their HCP to participate in the trial. Of these patients, 890 (17.1%) agreed to participate in the trial and 753 (14.5%) actually participated, 286 patients (5.5%) declined participation, and 4005 patients (77.0%) did not respond at all. Three hundred and 31 (44.0%) of the participating patients were randomised to the intervention group and thus received the intervention, and were invited to participate in this process evaluation. Two hundred and 14 (64.7%) patients from the intervention group filled in the evaluation questionnaire. Table 3 shows the characteristics of these patients. Demographic characteristics of patients that did not respond to the evaluation questionnaire (*n* = 117) were evaluated to compare with demographic characteristics of the responders. Non-responders were on average 51.9 years old (SD 14.4), mostly female (*n* = 73), native Dutch (*n* = 94), and had a background in higher (*n* = 57) or vocational educational (*n* = 31).

**Table 3:** Characteristics of patients that completed evaluation questionnaire (*n* = 214)

Mean age (SD)	56 (13.5)		Occupational status (%)	
Gender (%)	Male	Female	Student	7 (3.3)
	101 (47.6)	111 (52.4)	Employed	94 (44.3)
			Self-Employed	33 (15.6)
Back pain (%)^a^	124 (57.9)		Unemployed	36 (17)
Mean Disability Score (SD)^b^	4 (4)		Retired	42 (19.8)
Disability pension (%)	11 (5.2)			
			Volunteer work	26 (12.3)
Level of education (%)				
None/Elementary	6 (2.8)		Ethnicity	
High School	14 (6.6)		Native Dutch	198 (93.4)
Vocational	55 (25.9)		Western ethnic minority	8 (3.8)
Higher	137 (64.6)		Non-western ethnic minority	6 (2.8)

^a^N patients that reported having LBP at start of the study

^b^As measured with Roland Morris Disability Questionnaire [41], scale range 0–24

#### Dose delivered

Eleven out of 12 video-messages were uploaded according to protocol, thus receiving 11 points for this component of the dose delivered. Out of 24 monthly newsletters, 17 newsletters were sent according to protocol, and 7 newsletters were not. Thus, the newsletters scored 17 points, making the total dose delivered points amount to 28 out of 36 points (77.8% overall dose delivered).

#### Dose received and Satisfaction

Eleven (5.2%) patients stated that the website was recommended by their HCP, and 4 (1.9%) patients stated that they discussed the content of the website with their HCP. The Facebook page had 9 followers, and the Twitter account had 14 followers. Two patients stated that they used Facebook, and 1 patient stated using the forum. None of the patients indicated using Twitter. The monthly newsletters were considered a little useful by 137 (66.2%) patients, and not useful to 70 (33.8%) patients. The website login log showed that a total of 302 logins were registered, belonging to 170 unique patients (55% of intervention group). One hundred patients (71.4%) stated that they would recommend the website to others, and the mean satisfaction with the website was 6.7. Table 4 shows other results of the evaluation questionnaire. The majority of patients only visited the website once or not at all. More than half of the patients (53%) did not watch any of the videos. Patients who watched the videos seemed to appreciate the video-messages to an ample degree (graded 6.9 out of 10 points), but the majority felt little added value of the website to their recovery.

**Table 4:** Results of evaluation questionnaire (*n* = 214)

Website visits n (%)		Website clear n (%)	
Never	66 (31.3)	No	2 (1.4)
Once	91 (42.9)	Little	15 (10.5)
At least once per month	14 (6.6)	Yes	126 (88.1)
At least once, not every month	41 (19.3)		
Website useful n (%)		Added value of website n (%)	
No	11 (7.7)	No	44 (30.8)
Little	56 (39.2)	Little	61 (42.7)
Yes	76 (53.2)	Yes	38 (26.6)
Videos viewed n (%)		Videos useful n (%)	
None	76 (53.1)	No	12 (18.5)
One	26 (18.2)	Little	53 (81.5)
Some (2–12)	39 (27.3)	Yes	0 (00.0)
All	2 (1.4)	Mean appreciation videos (SD)	6.9 (1.3)
Links viewed n (%)		Links useful n (%)	
Never	75 (53.2)	No	5 (7.5)
Once	44 (31.2)	Little	61 (92.4)
More than once	22 (15.6)	Yes	0 (00.0)
Exercises viewed n (%)		Exercises useful n (%)	
Never	63 (44.7)	No	12 (15.4)
Once	31 (22)	Little	66 (84.6)
More than once	47 (33.3)	Yes	0 (00.0)
Contribution to recovery n (%)	22 (15.6)	Advice applicable	
Information	11	No	36 (25.5)
Videos	4	Little	63 (44.7)
Links	2	Yes	42 (29.8)
Exercises	19		
Social media	2		

Statistical analysis showed that patients who had not visited the website at all or only visited it once, rated the intervention with 6.4 points on average. Increasing the number of visits to monthly visits, or regular visits that were not per se monthly, resulted in an increase of average rating of 0.8 points, and the increase was statistically significant for patients that regularly visited the website. Table 5 shows the results of this linear regression analysis.

**Table 5:** Results of linear regression analysis on intervention rating

Group	B	Sig. (*p = .05)*	95% CI
Website visited 0 or 1 times (*n* = 157, 74.2%)	6.4	-	6.1–6.7
Website visited monthly (*n* = 14, 6.6%)	7.2	>0.100	6.0–8.3
Website visited regularly (*n* = 41, 19.3%)	7.2	<0.001	6.3–8.0

### Qualitative results (satisfaction, barriers and facilitators)

Forty-four semi-structured, qualitative interviews were conducted among patients with LBP, of which 19 participated in the entire study, and 25 only participated in this process evaluation. Fifteen patients were of Dutch or other western origin (mean age 57), 9 were of Moroccan origin (mean age 44.3), 10 were of Surinamese or Indonesian origin (mean age 34.6), and 10 were of Turkish or Iraqi origin (mean age 37.6). The overall mean age of the patients was 45 years, 25 were female and 18 were male. The majority of patients (*n* = 25) had a high educational level, followed by 15 patients with vocational education, and 4 patients with elementary education. The characteristics of these 44 patients are shown in Table 6. Data of the interviews were analysed and categorised into four themes, discussed by theme below.

**Table 6:** Characteristics of patients that participated in the qualitative evaluation

ID	Age	Gender	Ethnicity	Educational level	Study participant	ID	Age	Gender	Ethnicity	Educational level	Study participant
1	30	F	Polish	Higher	√	23	29	F	Iraqi	Higher	-
2	55	M	Moroccan	Elementary	√	24	42	F	Moroccan	Vocational	-
3	63	M	Moroccan	Vocational	√	25	26	M	Moroccan	Vocational	-
4	55	F	Peruvian	Elementary	√	26	18	F	Turkish	Vocational	-
5	50	M	Surinamese	Higher	√	27	41	F	Indonesian	Higher	-
6	38	F	Surinamese	Vocational	√	28	27	F	Surinamese	Vocational	-
7	42	M	British	Vocational	√	29	44	F	Moroccan	Higher	-
8	65	M	Moroccan	Higher	√	30	32	F	Turkish	Higher	-
9	66	M	Dutch	Higher	√	31	25	F	Surinamese	Vocational	-
10	28	F	German	Higher	√	32	76	M	Dutch	Higher	√
11	73	F	Dutch	Higher	√	33	60	F	Dutch	Higher	√
12	59	M	Turkish	Higher	-	34	65	M	Dutch	Higher	√
13	46	M	Moroccan	Elementary	-	35	74	F	Swiss	Higher	√
14	38	M	Dutch	Higher	-	36	56	F	Dutch	Vocational	√
15	45	F	Iraqi	Vocational	-	37	58	F	Swiss	Higher	√
16	34	F	Surinamese	Vocational	-	38	64	M	Dutch	Higher	√
17	26	F	Moroccan	Higher	-	39	70	M	Dutch	Higher	√
18	55	F	Turkish	Vocational	-	40	26	F	Turkish	Higher	-
19	32	M	Moroccan	Higher	-	41	42	F	Turkish	Elementary	-
20	56	M	Surinamese	Vocational	-	42	48	M	Surinamese	Vocational	-
21	22	M	Turkish	Higher	-	43	34	F	Surinamese	Higher	-
22	18	F	Turkish	Vocational	-	44	23	F	Surinamese	Higher	-

#### Satisfaction with intervention components

The information on the website was appreciated by most of the patients. The website was considered to be clear and understandable, although somewhat basic. Patients indicated that the amount of information on the website was satisfactory, and most patients felt that their expectations were met by the website. Some patients even felt that there was too much (especially written) information on the website. The content of the website was perceived to be interesting and helpful by most patients, although they indicated that the website would have been more useful to them if had they received access at the start of their first episode of LBP, when they did not have much information about and experience with LBP yet. One patient mentioned that this was a reason to drop out of the study by stating: *“… I thought: Been there, done that. And that’s where I quit.” (Patient 6).*

Although for many patients the exercises provided on the website were not new (having received them from healthcare providers on earlier occasions), the exercises were perceived to be the most helpful and interesting of all components by most patients. The ability to look up the exercises at any time of the day, to always have instructions at hand, and to be reminded to exercise were deemed positive effects of providing exercises on the website. Some patients mentioned they would have appreciated additional and more specific instructions regarding the exercises, such as an overview of when and which effects on the LBP should be expected when certain exercises are performed, and how often and how intensive the exercises should be performed. For example, one participant would have liked to know *“…Which muscle groups you train when you perform certain exercises, and why this is good for your back.” (Patient 20).*

Most patients did not look at the provided links to other websites and additional information. The most frequently provided explanation for this was the perceived unnecessity of those links: patients felt that they had learned enough from the website alone, or they already had all the information they wanted before they visited the website. Patients that had already recovered from LBP felt no need for (additional) information. Patients indicated that they already knew enough about LBP and they stated that they preferred all available information in one place, so that looking up information is more convenient and less time-consuming. As one patient stated regarding the convenience of having all information in one place: *“You should be careful with providing too many links, for one could not see the forest for the trees anymore.” (Patient 43).*

Patients were satisfied with the option to download material from the website to their computer, mainly because they could print this information and then have it available off-line and in other formats. This was beneficial for patients if they were not able to use their computer for a prolonged period of time, as one patient illustrated: *“I just print it. Reading on the computer is a bit difficult for me sometimes, because I have a cataract.” (Patient 8).* Patients mostly downloaded and printed the exercises from the website, although the download materials were overall not often used.

The newsletters’ most common effect was reminding patients to visit the website, or to (re)start following advice they read on the website or received from their healthcare provider. One patient said: *“It is a nice reminder for me to take a look at the website again. And I like reading updates about the study.” (Patient 35).* Also, the newsletters triggered patients to think about their LBP and their current state of health, and the patients appreciated reading about new information, insights, and updates from the research team. Since the patients received the newsletters directly to their e-mail, most could open the letter on their mobile device and thus read it at their convenience. This direct and approachable way of communicating information was appreciated by most patients, as *Patient 24* illustrated by saying: *“You get the newsletter in your mailbox and you can download and read it immediately. It’s no trouble at all. And I think it is interesting to read about what is going on.”* However, some patients indicated that they would have preferred another frequency of the newsletters (i.e. either more often or less often), and that they would appreciate more information about international research on LBP, for example on the aetiology and possible treatment options of LBP.

Many patients did not watch (all of) the provided video-messages due to time constraints, too much information on the website or technical issues with the website. Those that did watch the video-messages considered them informative, clear, and concise. The videos were considered easily accessible, and those patients appreciated the fact that the information was provided in a concise and present-day manner. They most often watched the videos in which a healthcare professional was interviewed; the videos with LBP patients were less often watched. The patients that watched the videos considered them to be informative, because they recognized their complaints in the stories told, and this made them more confident that their LBP is normal, and that medical interventions were not necessary. Even some patients that did not recognize their complaints in the video-messages considered the videos to be informative, because the stories reassured them and made them think more positively about their LBP and recovery. The patients mentioned that the experiences of other patients with LBP motivated them to actively work on their LBP, and gave them hope that recovery from their LBP is possible, as one participant stated: *“It is good to hear from someone that has gone through this in his life, and who really has gotten better. It gives you more willpower to do it yourself as well.” (Patient 26).*

The majority of patients did not notice the option to connect to the research on social media. Those patients that did use social media appreciated this option, because it made the information even more readily available and accessible. However, they also considered that the campaign was not active enough on social media. Furthermore, some patients indicated that they preferred social media over a website, because it is more interactive, allows for easier contact and information sharing with professionals and other patients. One patient stated: *“Facebook is more effective than a website. All the information is just there when you open it. … You can read it or take action.” (Patient 12).* Some patients were less interested to use social media, because they doubted confidentiality and reliability of the information provided and shared, but mostly because they did not use social media at all. Overall, ‘open’ social media (e.g., Facebook) was preferred over ‘closed’ social media (e.g., forum on protected login website).

#### Perceived benefits of intervention

Patients stated that they experienced various benefits of the website. For example, they noted that the information provided was reassuring, increasing their knowledge, providing insight and awareness, and improving their mental attitude about their LBP (e.g. by hearing about others’ experiences with LBP). The website was seen as a second opinion for the information patients had already received from their healthcare provider, and in that way the information was either complementary to their treatment, or a reminder for the information they had already received. Patients also felt that the website alerted them to the importance of exercise in LBP recovery, and it motivated them to start exercising. Equally important to the patients was the fact that the website was always available if they wanted to look up exercises or other sorts of information, and they felt that their LBP was taken seriously. One participant stated: *“Imagine that the physio has no time for you, then you can do the exercise at home. In the evening or at any time, and that’s great. So that is definitely the added value.” (Patient 23).*

In order to perceive the benefits of the website, patients indicated that it was important that they trusted the information on the website. Patients also stated that the opinion of their healthcare provider about this website was important to them: if he/she refers a patient to the website, the website is perceived as trustworthy and helpful, leading to an increased number of visits to the website. Involvement of the healthcare provider led to increased perceived trustworthiness and use of the website.

#### Usage of intervention

The majority of patients stated that they visited the website only once, and the visits usually lasted 10 to 30 min. The main reason for patients not to return to the website was that they did not experience LBP anymore, and patients with chronic or recurring LBP were already familiar with the information provided on the website. Some patients also noted that the information on the website was not applicable to their personal situation: *“There were a lot of things for me that I already knew or tried, that don’t apply to me, or I knew that they would not help me.” (Patient 24)*. Other barriers for usage of the website were not remembering to visit the website, a lack of time, difficulty with the language, and dissatisfaction with the medium used.

Patients that indicated visiting the website repeatedly mentioned experiencing several triggers for returning. Most often, receiving the monthly newsletter reminded them of the website and stimulated a return visit. Another important trigger was the need to refresh their memory about the exercises or other information, such as tips and tricks to reduce pain, or to see if any new information was available.

#### Satisfaction with medium used

Many patients indicated that their dissatisfaction with the medium used was a barrier for repeated or regular use of the website. Several components of the medium attributed to this, of which the layout of the website was one. Patients indicated that the website was perceived to be functional, but it was not attractive and did not draw attention, because of its design and structure. Another hindering component was the usability of the website. The website was not entirely responsive on some mobile devices, leading to discontinued visits in these cases. Many patients also indicated the necessity of protected login to be a barrier for visiting the website. They often forgot or lost their login credentials, and noted that logging in limited them in visiting the website, and sharing information from the website with others who did not participate in this study. This also led to discontinued visits and the preference of other, non-protected websites for information, illustrated by one participant by stating: *“It [login] obstructed me. I wanted to visit the website, so I looked it [login credentials] up, but actually I wanted to drop out because of it.” (Patient 28).*

While most patients indicated that they were content with the information provided via the website, some patients would prefer additional facilities. These included personalised information, more frequent and instant triggers and reminders (e.g. push notifications), and the possibility to directly connect with a healthcare professional. Most non-native respondents appreciated the translated parts of the website, and indicated that translations were important to involve a broader target group of patients, e.g. ethnic minorities who do not understand the Dutch language. These translations made these patients feel welcomed and valued, which increased their willingness to participate in the study, to visit the website, and to make use of the information provided. For the translations to be even more helpful, patients indicated that they should be translated professionally, into more languages/dialects, and, most importantly, that all components of the intervention should be fully translated.

### Comparison between groups

The satisfaction and experiences with the content of the intervention did not vary much between the various ethnic groups. Non-western patients (i.e. from Moroccan, Turkish/Iraqi or Surinamese/Indonesian origin) were more often not satisfied with the medium used, and indicated that they would have preferred a more modern, quick and on-the-go approach to information transfer than a website, for example using a mobile app that they could consult easier and more often. Non-western patient also viewed the exercises more often, and attached more importance to the translation of strategy materials than native Dutch patients.

## Discussion

This process evaluation showed that the protocolled strategy components were performed as planned to a dose delivered degree of 78%. Login registration data showed that 170 unique patients (55% of intervention group) logged on to the website, while a total of 302 logins was registered (1.8 visits per patient on average). Self-reported usage of the website (dose received) by patients showed that most patient only logged in once (*n* = 91, 42.9%). Although the majority of the patients who logged in did not view a large part of the website (i.e. video-messages, links, exercises), 70% (*n* = 99) of those patients perceived the website of added value and would recommend it to others (*n* = 100). Patients’ overall rating of the website was reasonable (6.7 out of 10). However many barriers for use, including information saturation and lack of translations, were identified in this evaluation. The reach of patients for this study was low, with only 14.1% (*n* = 753) participating patients.

### Interpretation of findings and comparison to other studies

The low reach found in the present study is in line with other implementation studies, for example that performed by Tonnon et al. [17], where the implementation of a lifestyle intervention in a workplace setting reached only 2.4% of the target population. The reach of the current study was not as high as for example the study performed by Buist et al., where the reach was 22.4% in an integrated healthcare system [18]. It must be noted that assessing the actual reach of a target population in implementation research is hard to measure, as is also discussed by Van Vilsteren et al. in their process evaluation of a workplace intervention for workers with rheumatoid arthritis [19]. For example, the amount of patients that were invited to participate could have been an overestimation, since the HCPs inviting the patients could have excluded patients without reporting exclusions to the research team. The reach of this implementation study can be compared to awareness levels of back pain mass media campaigns that have been conducted in other countries. For example, an effective Australian campaign reported that up to 86% of survey respondents reported having seen the mass media campaign [20]. While this is a high percentage, similar studies from Norway, Canada, and Scotland have reported awareness levels of 40, 50, and 60% respectively [21–23].

The results of this study suggest several possible reasons for the low use of the intervention. One explanation could be the quick recovery from LBP by some patients, after one visit to the website, or even before the visit. Another barrier might be the fact that the HCPs rarely discussed the website with their patients, and slightly more patients, although only 5%, were actually referred to the website by their HCP. This is supported by the qualitative results, in which patients indicated that referral to the website by their HCP leads them to trust the website more. This implicates that eHealth interventions should be blended in usual care, so called blended eHealth intervention. Thereby, involvement of HCPs may increase use of the campaign. These findings are in line with other studies in comparable primary care settings, for example a study performed by De Jong et al. [24], who offered a web-based counselling program for employees on sick leave due to non-specific low back pain or neck pain, and their occupational physicians (OPs). Although their participants appreciated the program, actual program utilization by the employees as well as by the OPs was low in the study by De Jong [24]. However, the low use in the current study was not in line with the aims and expectations of the intervention providers. Since the HCPs also received a multifaceted implementation strategy, and played an important role in including patients for the study, it was expected that they would play a bigger role in activating patients to use the eHealth campaign [13]. A literature review on public engagement with eHealth has suggested that health professionals play an important role in endorsement and promotion of eHealth services to patients [25]. The same review also suggested that trust might influence patients’ perception of eHealth services, for example concerns about scientific sources of the information provided [25]. An observational study on self-management of chronic neck and LBP showed that adherence to non-pharmacologic self-management strategies increased when patients received information about their illness and the effectiveness of the self-management strategy during the clinical course from their HCP [26]. From the current study it seems that involvement of the HCP and the perceived trustworthiness of the eHealth service may be related and their combination could be a factor in the use of the eHealth campaign. Unfortunately, both barriers seem to have influenced the use of this campaign in the current study.

Another barrier for usage of the eHealth campaign might be the dissatisfaction with the content and layout of the website. Many patients indicated that the information provided on the website was already known to them, and that the layout and design of the website did not trigger return visits. This is in line with the importance of both content and design of eHealth intervention that has been shown in previous studies [27, 28]. It seems not only important that patients receive the intervention (i.e. the information) in a timely manner (at the start of their first episode of LBP), but that the information also be communicated in a state-of-the-art manner. Designing eHealth intervention with high levels of interactivity in an interpersonal, dynamic, and engaging digital environment seems to be an important aspect in increasing the usage and effectiveness of eHealth interventions [24, 28, 29]. In the current study, mainly non-western immigrant patients were dissatisfied with the medium used. This may be explained by the difference in mean age and gender of the patients. Non-western immigrant patients were on average 18 years younger (mean age 38.8 years) than native or western-immigrant patients, and were mostly female (*n* = 18). The Centres for Disease Control and Prevention have shown that women are more likely than men to use the Internet for health information [30]. National statistics from the United States and the United Kingdom have shown that Internet use among 15–44 year olds is highest, and that usage decreases with the increase of age [31, 32], although there is no evidence that this is true for health information seeking specifically. Research has also shown that health care utilisation is higher among the immigrant population than among the native Dutch population [33–35], and that ethnicity is a predictor for health care utilisation regardless of health status [35, 36]. It also seems that immigrant patients with LBP have a worse prognosis than their native Dutch counterparts [37]. A possible explanation for these differences between ethnic groups might be that health care interventions insufficiently take the various needs and perspectives of immigrant patients into account. Therefore, to better cater to the needs of this population group, it is important to account for these ethnic differences. This could be done by targeting health care interventions specifically to this population and their beliefs.

For non-western immigrant patients, the lack of full translation of all intervention components also seemed to be an important barrier for intervention use. By translating the most important items of the intervention (i.e. the video-messages and home page information), the current study aimed to actively involve these patients. Although it might seem common sense to ensure that interventions are linguistically understandable to a broad group of patients, research has shown that it is not the only important aspect playing a role in involvement of culturally diverse patients [38, 39]. In all phases of the research process, from recruitment through staff that reflects cultural diversity to the use of linguistically and culturally appropriate materials, it is important to dwell upon the needs of various patient groups in order to involve them in health research and intervention. Assessing the needs of these patients prior to the development of healthcare interventions might increase the usefulness and effectiveness of these interventions.

### Strengths and Limitations

When interpreting the results of this study, some limitations have to be taken into account. Although effort was put into the recruitment of LBP patients with non-western ethnic backgrounds (especially Turkish, Moroccan, and Surinamese patients) in the trial, most participating patients were native Dutch or western immigrants. This led to ad-hoc recruitment of these ethnic patients for the sole purpose of the current process evaluation. The patients in the qualitative evaluation only received access to parts of the intervention (i.e. the website and social media), and thus did not have the full-experience of the entire intervention. This might have influenced their satisfaction and experiences with the website. Furthermore, it is plausible that illiterate and thus low or non-educated respondents were underrepresented in this study. Another notable finding in this study was that the majority of patients had a high educational level. Although this is in line with other research [40], it is important to put effort into the recruitment and participation of lower educated patients. The fact that the majority of patients that participated in the quantitative process evaluation had a high educational background might have influenced the results, as these patients may have different needs and opinions than lower educated patients. Also, the dose delivered component should be interpreted with caution, as it is an arbitrary quantification of parts of the full strategy, and has its own limitations. For example, due to planning issues, only 11 out of 12 video-messages, and 17 out of 24 newsletters were delivered on the planned date.

This is also true for the dose received, which was defined as the proportion of patients who logged into the website at least once. Whether these measures reflect effective doses is debatable, but it makes a first step in increasing the quality of the current study. Ideally, if technical opportunities allow for this, intervention providers should collect data on actual use, for example by tracking time spent on the website or website components used. To further increase the quality of this process evaluation, a mixed methods approach to data collection and analysis was applied. Triangulation of these methods (i.e. descriptive quantitative analysis, regression modelling, and qualitative data analysis) improved the quality of this evaluation. Furthermore, to ensure correct analysis and interpretation of qualitative data, a large random sample of all interviews were coded and discussed by two independent researchers.

### Implication of findings

The results of this study showed that many patients did not use the intervention at all or only once, and this is an important finding in the light of the future effect evaluation of this implementation study. As this patient based eHealth campaign was part of a larger implementation study, it was designed to support the HCP based implementation of a guideline that advocates reduced referral rates for diagnostic imaging and consultations with medical specialists [13]. The current process evaluation showed that HCPs rarely discussed the eHealth campaign with their patients, indicating that the patient based and the HCP based campaigns were too independent of each other. This may be reflected in the final results of the implementation study (i.e. reduced referral rates for LBP). HCPs should discuss with and stimulate the use of eHealth interventions by their patients, and implementation strategies that are targeted at changing patient outcomes should ideally also pay more attention in their strategy to the improvement of HCP behaviour and attitude towards these interventions.

## Conclusion

This process evaluation showed that the multifaceted implementation strategy was used by only a small proportion of the patients with non-specific LBP. It seemed that the campaign was offered to the patients too late, that the lay-out of the campaign did not meet the needs of the patients, and that healthcare providers rarely discussed this campaign with the patients, while involvement of those providers seemed to improve trustworthiness of the campaign and increase its usage. As the current study showed that the satisfaction of the intervention increased with more visits to the website, it probably pays off to invest in motivating HCPs to activate patients to use eHealth interventions at the right timing (i.e. at patients’ initial consultation with the HCP). Needs assessment research might contribute to the development of (more) serviceable eHealth interventions. Future researchers and practitioners may benefit from including patient perspectives and expectations, and adapting interventions to the targeted population in terms of language, content, and delivery method, while health care providers could aid in promotion of eHealth interventions and self-management.

## Abbreviations

CME: Continuing medical education
HCP: Healthcare provider
LBP: Low back pain
OP: Occupational physician
RCT: Randomized controlled trial
